# Images, Words, and Imagination: Accessible Descriptions to Support Blind and Low Vision Art Exploration and Engagement

**DOI:** 10.3390/jimaging10010026

**Published:** 2024-01-18

**Authors:** Stacy A. Doore, David Istrati, Chenchang Xu, Yixuan Qiu, Anais Sarrazin, Nicholas A. Giudice

**Affiliations:** 1 INSITE Lab, Department of Computer Science, Colby College, Waterville, ME 04901, USA; dcistr26@colby.edu; 2 Department of Computer Science, Fu Foundation School of Engineering and Applied Science, Columbia University, New York, NY 10027, USA; cx2295@columbia.edu; 3 John A. Paulson School of Engineering and Applied Sciences, Harvard University, Allston, MA 02134, USA; 4 Sonos, San Francisco, CA 94109, USA; anais.sarrazin@sonos.com; 5 VEMI Lab, School of Computing and Information Science, University of Maine, Orono, ME 04469, USA

**Keywords:** assistive technologies, image captioning, accessibility

## Abstract

The lack of accessible information conveyed by descriptions of art images presents significant barriers for people with blindness and low vision (BLV) to engage with visual artwork. Most museums are not able to easily provide accessible image descriptions for BLV visitors to build a mental representation of artwork due to vastness of collections, limitations of curator training, and current measures for what constitutes effective automated captions. This paper reports on the results of two studies investigating the types of information that should be included to provide high-quality accessible artwork descriptions based on input from BLV description evaluators. We report on: (1) a qualitative study asking BLV participants for their preferences for layered description characteristics; and (2) an evaluation of several current models for image captioning as applied to an artwork image dataset. We then provide recommendations for researchers working on accessible image captioning and museum engagement applications through a focus on spatial information access strategies.

## 1. Introduction

As venues fostering intellectual curiosity, museums and online art collections can create complex intellectual interchanges between the visitor and the exhibit curator through independent exploration, engagement, and close study. However, fully engaging with visual artwork in these settings presents significant challenges for visitors with blindness and low vision (BLV). While many museums provide art placards, audio information guides, and online alt-text descriptions that provide some information about the artist, the provenance, and the medium of the artwork, these descriptions rarely provide enough information for the BLV visitor to truly understand what is depicted through the formation of a mental representation of the artwork. Museums often lack the resources needed to create these accessible descriptions and the dynamic nature of art installations can make it difficult for curators to develop fully inclusive museum exhibits. There has been a growing body of research attempting to leverage recent large language models (LLMs) with computer vision to create general audience artwork descriptions. However, few of the automated captioning models have been evaluated to determine if the captions generated meet the needs of the BLV community. This paper describes two studies investigating the type of information needed to support accessible artwork descriptions. In the first study, BLV participants identified language characteristics necessary to form mental models of artwork. In the second study, we evaluated the capacity of four image captioning approaches to generate artwork descriptions on both the COCO dataset [1] for image captioning, and on the SemArt dataset [2] which contains copyright free classical artwork images from the Web Gallery of Art (WGA) [3]. The model evaluation results will highlight the challenges of using many of the current approaches in the domain of image captioning for BLV audiences. In summary, our main contributions are as follows:

- We offer a combined methodological approach of collecting user-centered captioning feedback from BLV participants with a comparative image captioning model evaluation.
- We provide evidence that expert user feedback should inform the structure of image captioning evaluation and model testing within a human/model collaborative framework. Accessible captions should not be a specialized domain.

We argue that this study has important elements that most imaging studies do not—comparing user-centered design for a specific target audience with a technical model evaluation. Most other papers on this topic (i.e., captioning for BLV individuals) are either exclusively user-driven or image caption model driven. We believe this is a critical methodological step and that using real user data to guide the structure of the extended descriptions that are most relevant helps to ground the user requirements research within the model evaluation. This human/model collaboration is a unique component of the work and a novel contribution to the literature as a research approach that we advocate for being adopted in similar studies moving forward.

The paper begins with an overview of related research in the areas of generating accessible descriptions within the domain of art images, both through expert curators and automated captions. We then present a brief summary of current deep learning image captioning methods, highlighting the models that are used in the later evaluation study. The qualitative user study and the model evaluation study follow, with a discussion of the findings.

## 2. Related Work

### 2.1. Accessible Descriptions

There have been recent improvements to make museum collections more accessible for BLV individuals, including providing enhanced descriptions about gallery exhibit objects [4,5,6,7,8]. Accessible art description guidelines, such as those created by the Art Beyond Sight (ABS) project [9,10], help curators create standardized descriptions to assist with non-visual art interpretation. This framework prioritizes language to facilitate the building of robust mental models of art objects for BLV audiences [11,12,13]. The guidelines recommend the use of consistent terms, rules for information ordering, and spatial concept presentation which can help the listener to focus attention on the elements that are most salient to the image, while also reducing the cognitive load that is inherent in the use of longer verbal descriptions [14,15].

Several museums have used online portals to create crowd-sourced descriptions of artwork developed by museum curators for BLV audiences [16]. This curatorial approach often involves partnerships, such as joint work conducted by the Chicago’s Museum of Contemporary Art (MCA) and a group of accessibility researchers [17]. Based on earlier ABS recommendations, additional MCA guidelines were created to provide a basic framework for museums to develop more effective non-visual descriptions that consisted of both short and long descriptions. The short overview description (i.e., Alt-text) was a one sentence caption with a maximum length of no more than 10 words [17]. The long description could be extended up to a paragraph and was intended to fully capture detailed information that would help a BLV individual formulate a mental representation of the artwork [6]. While these curator generated accessible descriptions provided opportunities for improved artwork engagement experiences for BLV visitors, there were several remaining shortcomings. They did not explicitly convey the kind of spatial information that would be critical for a BLV visitor to form a mental map of the artwork, nor did they incorporate the large body of research on how humans conceptualize spatial scenes through language as opposed to vision [18,19,20,21]. Effective accessible image captions should describe salient regions of an image comprehensively and concisely in the form of well-structured and linguistically expressive sentences. Combining expert knowledge, current research on accessible descriptions, and automated image captioning techniques could help to streamline the process, increasing access to art images for the BLV community.

### 2.2. Automated Image Captions

Automated image captioning, the task of describing visual images in natural language with syntactically correct and semantically meaningful sentences, requires both a visual understanding system and a generative language model [22]. Most captioning systems for artwork do not include specific considerations for what might be required for non-visual mental representations of artwork for accessible BLV engagement [23]. A full review of deep learning image captioning approaches is beyond the scope and purpose of this paper, however, it is useful to provide a brief overview of current image captioning frameworks that are the focus of the models used in the evaluation study (Section 4), based on their application to the domain of automated accessible descriptions for BLV audiences.

Traditional deep learning methods using a combination of Convolutional Neural Networks (CNN) and Recurrent Neural Networks (RNNs) have been applied to the captioning of artwork image datasets, painting style classification [1,24,25,26,27] or visual feature search and retrieval within the paintings [28,29]. However, models trained using real world image training datasets have often introduced a gap between the source domain (photographs) and the target domain (artwork) at training and testing [30,31]. To date, progress on image captioning specifically for artwork images has been limited by the resource intensive process of creating training datasets of sufficient size and annotation detail [2]. Recent advancements have begun to address this domain gap, meaning that it is worth reviewing the different approaches employing deep learning whole image captioning methods. These approaches are generally classified around the steps in the task (e.g., visual encoding, language modeling, and training) and their core structures [32]. This includes a number of broad categories of image captioning approaches [22,33]: non-attention-based, attention-based, graph-based, transformer-based (self-attention-based), and vision language pre-training (VLP), as well as a number of hybrid variations of these approaches.

#### 2.2.1. Attention-Based Approaches

While there are many variations of attention-based captioning methods, they are generally based on theories of human attention, salience patterns, and optical focus. During the model training process, the model is supervised to focus on specific regions that utilize cues to highlight the most salient parts of the image for generating captions. Traditionally, a CNN or a region-based CNN is used in the encoding stage for extracting the visual information and then an RNN generates a linguistically acceptable description in the decoding process. In some cases, actual human eye-fixation or predicted saliency data might guide the model in focusing on the most salient elements for caption generation [34,35]. While attention-based image captioning methods have produced improved accuracy and caption fluency over previous global CNN non-attention-based models, a significant downside of these approaches is that they do not capture information about the spatial relations between the objects detected inside the image. In order to address this shortcoming, some approaches have added spatial and semantic relations to an attention-based framework to focus attention on different image regions based on a set of conditions and dependencies between words creating trees that can be organized around noun-modifier chunks that are tied explicitly to image regions [36]. Based on the known attention-based method challenges, including the computational complexity associated with object detectors and the need for detailed bounding-box annotations, we have not included older non-attention or attention-based approaches in our evaluation study.

#### 2.2.2. Graph-Based Approaches

Many image captioning studies have turned to scene graphs and graph convolutional networks (GCN) to represent spatial and semantic relationships extracted from image regions and from textual data [37,38]. For example, Shi et al. [39] generated semantic graphs based on training the module on the ground-truth captions and the results were then input into a GCN used for both encoding and decoding processes. In a similar approach [40], scene graphs were developed using two processes (i.e., concept cognition and sentence reconstruction). The process generated spatial and semantic graphs through a CNN (visual feature extraction) to an RNN (relationships and dependencies), and a Support Vector Machine (SVM) (concept classification) pipeline which then were passed through the language model to generate the captions. Finer-grained spatial and semantic information can also be achieved through the identification of sub-graphs within the image combined with attention-based LSTM that generates the sentences [41]. Although these progressively more refined hybrid graph-based methods offered much in improved spatial and semantic relationship details, recent trends favor transformer-based and Vision Language pretraining models for their performance in handling multimodal tasks such as image captioning.

#### 2.2.3. Transformer-Based Approaches

Transformer-based approaches [42] have been applied to image captioning tasks using complex multiple layers and fully attentive encoder/decoder mechanisms that connect the image elements to the language model in combination with techniques for processing semantic knowledge [43,44,45]. This line of research produced the first convolution-free approach for image captioning [46], replacing the CNN image encoder with a pre-trained vision transformer network and combining it with a language transformer decoder. In this study, we evaluate the One-For-All method (OFA), [47], a recent unifying multimodal pre-training model that uses a sequence-to-sequence CNN and a transformer. Another model included in this study, ExpansionNet 2.0 [48], consists of the standard encoder/decoder structure implemented on top of the Swin-Transformer [49], however, it distributes the input over different length sequences.

#### 2.2.4. Vision Language Pre-Training Approaches

Vision language pre-training (VLP) models are the latest training method for image captioning tasks showing great promise and impressive performance. In VLP approaches, large-scale models (image and language) are pre-trained on massive datasets [50]. We focus on two of the current VLP methods. CLIP Prefix Caption [51] approaches the image captioning task through combining contrastive learning (CLIP [52]) with an integration of a pre-trained large language model (LLM) or GPT2 (Generative Pre-trained Transformer) [53]. The CLIP encoding is used as a prefix to the caption and the hybrid approach enables the model to effectively process and understand both visual and textual information while also offering the advantages of being a simpler, faster, and lightweight model. With the advent of publicly available LLM powered image captioning models, it made sense for us to include this in our evaluation of image captioning models as applied to artwork. The BLIP models [54,55], also featured in this paper, are based on a VLP framework with a pre-trained querying transformer learning from an image encoder and a generative language model.

We now turn our attention from a summary of image captioning approaches to a study of how the primary users (i.e., BLV individuals) of this information prioritize the information they need to interpret complex artwork scenes.

## 3. Materials and Methods

### 3.1. Study 1: Language Preferences for Image Mental Models

This first study was motivated by the need for more specific information from BLV individuals about the kinds of information and the level of detail needed to accurately access and interpret artwork through language alone. **(RQ1)** *What are the critical descriptive language structures that should be embedded into accessible automated artwork descriptions?* We hypothesized BLV participants would prefer descriptions that featured a higher degree of spatial language, e.g., reference boundaries, distance, orientation, and composition of the images. To test this hypothesis and the effectiveness of the current recommendations for accessible descriptions, we designed a small survey featuring five artwork images with a short overview and a long thematic description that had been developed by curators in the MCA Coyote project [56]. Five new ‘spatial’ descriptions were also created for the images that focused exclusively on spatial information such as boundary references (top/bottom, left/right, middle), orientation, object relationships (near, against, on top, underneath), number of objects and their configuration, as well as other relevant composition features.

### 3.2. Participants

We recruited 11 BLV participants (P1–P11) (Appendix A Table A1) who self-reported as legally blind through several announcements on BLV interest email listserves to evaluate the different descriptions in order to learn more about the specific types of language elements they preferred in visual artwork descriptions. To participate in the study, participants needed to be 18 years or older, be legally blind, have experience with going to museums or art galleries, and be able to communicate in English. Of the 11 participants we recruited (F = 6, age range 18–65), approximately half of the participants identified as congenitally blind from birth or had experienced vision loss as toddlers, and about half identified as having limited residual vision such as light/dark perception. Participants were compensated with a $50 Amazon gift card. The recruitment and study procedures were reviewed and approved by the Colby College Institutional Review Board.

### 3.3. Image Selection

Similar to a recent study conducted by Li et al. [13], we selected images that varied in composition as well as in subjects or objects depicted (Appendix A). For instance, Image 1 is a self portrait, featuring a single person painting themselves; Image 2 represents a traditional still life with multiple objects; Image 3 is an abstract painting of fruit in an abstract style; Image 4 is a photographic portrait of person in a natural outdoor environment; and Image 5 is an artistically composed photograph of multiple people in a built environment.

### 3.4. Spatial Description

A description was constructed for each image with spatial information (distance, orientation, configuration, etc.) vs. thematic information (colors, emotional affect, style, medium, etc.). Each spatial description was structured as a paragraph that described objects, activities/interactions, and environmental features in the artwork specifically referencing spatial characteristics of objects, boundaries, object relationships, and the overall composition of the artwork. For example, below is the spatial description that was created for Kerry James Marshall’s painting *Untitled (Painter)* [57] from the online collection of the Museum of Contemporary Art Chicago (MCA) [57]:

*A woman painter sits holding a paint palette in front of a large paint-by-numbers portrait of herself in a similar pose. In the vertical portrait, the woman in the foreground and the painting in the background make up three quarters of the entire picture. From the bottom left corner, there is the top of a chair that she is sitting on while she looks directly at the viewer. In the top left corner, behind the woman, there is a dark background. The woman’s body is centered in the painting. She is shown from mid-body, with her right arm holding a long paintbrush, which is pulling paint from a palette held in her left hand. In the background, starting at the midpoint to the right edge, is a paint-by-numbers portrait of her. Much of the portrait she is working on has not been filled in, except for some of the background and her jacket, which is similar to the one she is wearing while she is painting.*

This description approach is based on current recommendations for accessible descriptions in terms of order of information, length, and reflects previous work evaluating the information preferences for both sighted and BLV users [13,58,59] It is also consistent with research on the layering of information in nonvisual image descriptions with the spatial boundary reference language used in accessible multimodal interfaces, thus taking advantage of commonly used reference frameworks familiar to the BLV community [60].

### 3.5. Protocol Design

The protocol presented participants with three types of descriptions (a short overview, a spatial description, and a thematic description) for a single image. The visual image was not presented in the survey so as to not advantage the participants who had some residual vision. The descriptions and participant directions were designed to be read with a screen reader. After reading the three descriptions, participants were asked about their preferences and what were the characteristics of the descriptions that they found most useful or thought needed to be improved:

1. Which image description did you prefer, the short overview, spatial description or the thematic description?
  They were also asked:
2. What specific aspects make your preferred description effective?
3. Is there anything confusing in your preferred description that could be improved?
4. Why are the other descriptions less effective? How could they be improved?

The survey was distributed over email using a Microsoft Word document to provide easier accessibility over online forms or survey platforms. When the participant finished the survey, they returned the document to the research team via email. Consequently, each study participant reviewed the five image descriptions (5 × 3 descriptions = 15) and answered 20 open response questions. Participants also answered five open-ended questions about their demographics, visual impairment, and experience with museums. A total of 220 open responses related to the image descriptions were recorded from all of the 11 participants.

The protocol did not specify elements of the image descriptions that we wanted participants to address in their responses, such as subject (e.g., people, environment, activity), form (e.g., shape, line, color), context (e.g., history, emotion, spatial relations) as has been done with similar studies on BLV image descriptions [13]. We wanted to focus participant attention on spatial information and these other types of information tended to distribute spatial information among the categories. Emergent coding methods based on grounded theory [61] were used to identify general themes as core codes and then more specific codes were identified in the open responses. Written responses (n = 165) were aggregated by key themes across participants. A summary of these findings are presented below based on the identified themes.

### 3.6. Results by Description Type

**Figure 1:**
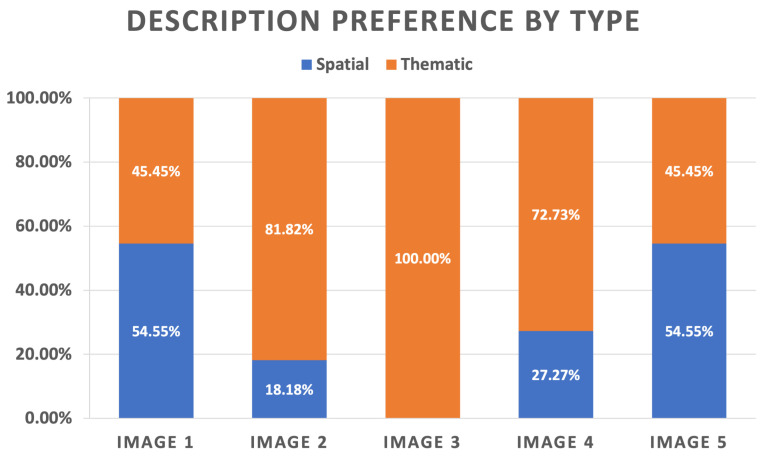
Selected Response Results of Participant Description Preference—Spatial or Thematic.

None of the participants reported that the short overview description was their preferred description and all reported their preferences for either the spatial or thematic descriptions for all of the images (Figure 1). Five images from the Museum of Contemporary Art Chicago (MCA) Coyote Project [56] were used in our interviews to examine the language and description elements that BLV participants preferred in artwork descriptions: Image 1 [57], Image 2 [62], Image 3 [63], Image 4 [64], Image 5 [65]. Between the spatial and thematic descriptions, a small majority of participants reported preferring the spatial description for Images 1 and 5 (Images 1 (55%) and 5 (55%)). A majority of participants indicated they preferred the thematic description for Images 2–4 (Image 2: 82%, Image 3: 100%, Image 4: 72%). Out of the total responses to the question about what could be improved in the image descriptions, 25% of responses specifically stated that the short overview descriptions were too short and did not contain enough detail to provide an accurate mental representation of the image content.

While on the surface, these selected response results appear to favor the thematic descriptions, or those captions that contained information about colors, emotional affect, and contextual significance. However, approximately 10% of those who reported preferring the thematic descriptions in the selected response questions specifically mentioned spatial information as the reason for their preference in the open response questions. Interestingly, there were more than double the number of comments about spatial information mentioned by participants who reported preferring a thematic description as compared to comments from participants that preferred the spatial descriptions (“*I liked how the description of how the orbs were arranged was easier to understand in the thematic description.*”). Just as there were participants that reported a preference for spatial information contained in the thematic descriptions, there were also a few participants that mentioned they enjoyed the description of colors in the spatial descriptions, although the spatial descriptions did not contain any references to colors. There are several possible interpretations of the blending of the different preferred types of information, including a preference for spatial information even is it is labeled thematic, a layering of information comprised on information within both descriptions, or participants mixing up what information was contained in each description.

### 3.7. Mental Imagery, Engagement, and Subject Details

The vast majority (70%) of responses about why participants preferred one description type over the other were related to specific details provided in the description images. Sometimes this was a general comment, with little explanation about the helpful details (“*It described the painting with just the right amount of detail.*”); in other cases, there was a specific type of detail that signaled spatial or thematic information (“*The other descriptions did not mention the branches and leaves and their proximity to the fruits*”). In many cases, participants reported being able to imagine the image in their minds using their preferred description, however, there were no significant differences between the type of preferred description and the ability to form a mental model. Both description types produced an equal number of comments (22%) about descriptions supporting mental imagery. There were the highest number of mentions of spatial information being helpful for mental imagery in responses for Image 1 and Image 4 (“*I could see the painting when reading/listening to the Spatial Description.*”, “*It gave me enough information to imagine what the painting looks like.*”, “*In the spatial description I could see the picture.*”). Both description categories produced a similar number of comments (spatial 35%, thematic 30%) about participants being engaged and eliciting positive feelings when hearing about the image. Most participants mentioned preferring one description over the other because of the amount of attention paid to describing subject(s) of the image. Subject details were frequently mentioned by those preferring thematic descriptions in Images 1, 2, and 4 (“*I preferred the thematic description because it described the types of clothes the woman was wearing*”), whereas in Images 2 and 5, the spatial descriptions were more frequently mentioned because of the details provided about the image subject(s) (“*I preferred the spatial description because it provided the types of fruits and sizes of items in full detail*”).

### 3.8. Image Form and Context

Regarding comments about image form and context, approximately 10% of the open ended responses mentioned that a description was preferred due to the spatial information (distance, orientation, configuration, composition, etc.) which provided context for where the image subject(s) were located and their relationships to one another (“*I liked the way the spatial description described the painting top to bottom and left to right and identifying the corners of the page. Knowing the location of the items helps me visualize the image.*”). The thematic descriptions had the largest number of comments about the importance of describing attributes of form, specifically colors, in the images (18%).

In summary, results from this study of layered image descriptions suggest that BLV participants preferred: (1) image descriptions that provided more information about the subject(s) than just a brief one sentence description, (2) they wanted detailed information that provided form and context of the image using spatial and thematic language, although which type of information was preferred depended on the image and the quality of the description, and (3) they wanted enough detailed information to be able to form a mental model of the image to increase their engagement with the artwork.

## 4. Study 2: Evaluation of Image Captioning Models for Accessible Artwork Descriptions

In the second study, we wanted to know: **(RQ2)** *Based on a selection of current image captioning approaches, which provides the most accessible artwork image descriptions?* We chose four currently available models transformer and VLP based methods that were trained using an artwork image dataset to evaluate the performance in generating accessible descriptions for art paintings. We hypothesized that models trained on an artwork image dataset would produce more detailed spatial language in the captions than those using large general purpose image datasets with natural images (i.e., photographs) and large language models.

### 4.1. Models and Dataset

The number of new image captioning models being released regularly made it difficult to select the most recent methods to use for this study, however, the models chosen either had a demonstrated strength in providing more details than other models (CLIP Prefix, BLIP) or had been previously applied to artwork images (Expansion Net, OFA). The CLIP Prefix Caption [51] model approaches the image captioning task through combining contrastive learning (CLIP [52]) with an integration of a pre-trained large language model (LLM) or GPT2 (Generative Pre-trained Transformer) [53]. The CLIP encoding is used as a prefix to the caption and the hybrid approach enables the model to effectively process and understand both visual and textual information while also offering the advantages of being a simpler, faster, and lightweight model. With the advent of publicly available LLM powered image captioning models, it made sense to evaluate its ability to generate captions for artwork. The BLIP model [54] is based on a Vision-Language Pre-training (VLP) framework which is designed for understanding and generation tasks. BLIP utilizes noisy web data by first generating synthetic captions and then filtering out the noisy ones. It has the advantage of performing well on a range of vision-language tasks, such as image-text retrieval, image captioning, and Vision-Question-Answer (VQA), and it is also able to be directly transferred to video-language tasks. The ExpansionNet [48] model uses an approach that processes the input across a heterogeneous and arbitrarily large collection of sequences with different lengths as compared to the input. The One for All (OFA) model [47], focuses on aggregating object-level features to enhance the caption generation process. This method has advantages when generating descriptions of complex art pieces, as it has the ability to capture intricate details and relationships among different elements within the image.

The SemArt dataset [2] was selected as the artwork image training dataset because it is one of the few collections of copyright-free artistic images (21,000) that have been annotated with rich textual descriptions. It was created using the Web Gallery of Art (WGA) [3], which contains more than 44,000 images of European fine-art reproductions created between 700 and 1800 CE. The WGA grants explicit permission for the collection images to be used in academic projects. The SemArt dataset is publicly available on the project’s github as it was specifically designed to facilitate machine learning research on semantic retrieval tasks that involve understanding visual arts and the relationship between images and natural language text. While the majority of the images are from western artists from the pre-1900s periods, it does feature a wide range of artwork images from various styles, periods, and artists. The image annotations or metadata contain the painting’s artist, artist’s birth and death, title, date, technique, current location, form, type, school and time-line.

### 4.2. Methods

The second study was focused on evaluating models that could maximize the amount of spatial language and details in each of the generated captions. The SpaCy library with the encore web trf model was used as part of the evaluation process based on the transformer architecture of RoBERTa-base [66]. This allowed for processing the generated descriptions using a standard natural language processing (NLP) workflow for analysis of the amount of specific linguistic structures such as (prepositions (relationships), adjectives (details) as well as nouns (objects) and verbs (actions) that denote the expressiveness of the generated captions. Descriptive statistics for the generated descriptions provide a more comprehensive understanding of caption length, variability, and overall quality of the generated captions based on each model’s strengths and weaknesses. BLEU-4 [67,68] and METEOR [69] scores were generated for the models using the COCO dataset [1] to generate a baseline comparison as well as on the SemArt [2] dataset for evaluating model performance on art paintings. BLEU-4 measures the n-gram overlap between the generated caption and the ground truth words in the captions, while METEOR assesses the semantic similarity between the generated caption and human written captions, taking into account both the content and the structure of the sentences. Using these common metrics on a dataset of art images, we assessed each of the models’ ability to generate high-quality captions that might provide detailed, accessible descriptions.

Four models (CLIP Prefix Caption, BLIP, ExpansionNet, and OFA), were tested for the capacity in transfer learning using an 80/20 train-test split of the dataset (Table 1). The results of the model evaluation are reported below, starting with a comparison of the descriptive statistics for the parts of speech contained in the generated captions for each model followed by a summary of the models’ performance based on BLEU-4 (word matching) and METEOR (semantic similarity) scores.

### 4.3. Study 2 Results

Each of the four models generated a one sentence description based on its prediction of what the caption should be.

Using standard caption quality measures (BLEU-4 and METEOR), the generated captions from the models were evaluated by comparing the performance on both a standard natural image dataset (i.e., photographs) (COCO) [1] as a baseline measure and the SemART dataset (artwork) [2] to determine if there was still a domain gap in the types of image content/style. The mean scores for each model are reported for the COCO dataset and the SemArt dataset (Table 2). Both measures evaluate the quality of a machine caption output by measuring its correlation to reference captions. The measures have been widely adopted because they are easy to understand and have demonstrated some correlation with human evaluation. The scores evaluate high quality caption results with a score of 1.0 to a low-quality captioning results with a score of 0.0. The closer the value is to 1, the better the predicted caption. While it is not possible to actually achieve a value of 1.0, a value higher than 0.3 is considered a good quality score for both measures [70]. BLEU-4 quality score is based on n-gram precision while METEOR is considered to be more advanced because also takes into account additional information such as synonyms, word forms, and sentence structure [71].

The results of the BLEU-4 and METEOR metrics for models’ the generated captions trained on the SemArt dataset (Table 3) suggest that based on a simple metric of n-gram matching (BLEU-4), most of the models, except for OFA, were able to achieve a similar average score (>0.30). This means that the words within the generated captions matched relatively well with the reference captions in the dataset. However, based on METEOR’s semantic similarity score results, none of the model generated captions for the SemArt test set were able to reach a similar moderate quality score as the generated caption content was not semantically similar to the SemArt reference annotations.

## 5. Discussion

In the first study, BLV participants were asked to evaluate three different descriptions created for an artistic painting image in order to explore the question: **(RQ1)** *What are the critical descriptive language structures or linguistic elements that should be embedded into accessible automated artwork descriptions?* Two of the descriptions in the study (short overview and thematic) were created by the curators in the Coyote project [17] and the spatial description was created by our research team. We hypothesized BLV participants would prefer descriptions that featured content that prioritized spatial language, (e.g., provided boundary, distance, orientation, and composition of the images) because it would help to formulate a mental model of the image more so than thematic information (e.g., colors, cultural/historical context, and artistic style). Based on the survey results, BLV participants reported they strongly preferred detailed captions that had both spatial information and thematic information. BLV participants strongly preferred image descriptions that provided more information about the image subject(s) than just a brief one sentence description (as are found in standard image captions). They also reported wanting art images descriptions with enough information to be able to form a mental model of the image and needing both types of information to do this effectively.

The thematic descriptions were written by MCA curators for the Coyote project as ‘long descriptions’, therefore, there were no restrictions on the kinds of information included in these descriptions, some of which was clearly spatial in nature. In contrast, the spatial descriptions were intentionally written to only contain information that was spatial in nature and were written specifically by the researchers for this study. There were a handful of participant responses preferring the spatial description because of the description of colors (thematic), however, the spatial descriptions did not actually contain description of colors. While this may be evidence of participants confusing description details in their responses, we argue that it may also be the effect of multiple layers of different types of information forming a single mental representation for these participants. This finding provides support for the use of layered descriptions that provide distinctly different types of information for mental image model building for BLV individuals. Although each type of description should be brief, the layering information, and perhaps the choice about which description might be prioritized in the ordering of the descriptions, could give BLV participants control over how they preferred to build up a mental model from the layering of image descriptions. This is in many ways similar to how people using vision engage with artwork in a museum setting to make meaning; focusing on distinct types of salient information of the artwork based on personal connections, part-whole arrangements and object configurations, artistic style, colors, and other visual features [72,73,74,75].

### 5.1. Comparison of Captioning Approaches

In the second study, four approaches for generating artwork image captions were evaluated to investigate the question: **(RQ2)** *Which of the current image captioning approaches provides the most accessible artwork image descriptions?* The concept of an accessible description, was defined as the generated descriptions with the greatest amount of spatial information and contextual details that would allow someone to form a mental image of the artwork, based only on the text description. We hypothesized that models trained on artwork image datasets would feature more spatial language in the descriptions than those using large general purpose image datasets and large language models. However, based on the preliminary results of the automated description methods compared in this study, none of the current models evaluated were able to produce the kind of rich descriptive language that would assist in forming an adequate mental model of the image.

The four models were effective in producing concise captions, identifying key elements, describing simple spatial relationships, and conveying actions or states. However, there was significant variance in the accuracy of the generated descriptions. While the BLIP model was effective in generating more accurate captions than the CLIP Prefix Caption model, it did not provide more spatial information about the image content. The generated captions, such as “a painting of a group of people and animals in a forest”, “a painting of boats in the water near a castle”, and “a painting of a man eating with three children”, are concise and accurate but lack the detail that would allow a BLV visitor to form a rich mental image of the artwork image. This limitation suggests that while these models were adept at identifying key elements in the artwork and simple statements about spatial relationships, they struggled to capture the full context of the image that would allow for meaning making and metal models of the artwork. On a positive note, the models could generate the short overview descriptions but our BLV participants did not find these to be helpful on their own. Our findings demonstrate that current models are not up to generating the detailed descriptions preferred by our BLV study participants. However, we believe the success in generating adequate short overview descriptions, coupled with better artwork image training datasets, and the continued advancement of generative AI approaches will lead to enough spatial information within image captioning models to eventually support BLV mental model building.

### 5.2. Mismatch of Accessible Information and Captioning Quality Metrics

Measures such as BLEU-4 and METEOR assess the quality of automated captioning models and are reliant on the matching of the reference sentence source of truth to the generated sentence. Based on the results of the evaluation using an average of the BLEU-4 scores for the generated captions in each of the models, all of the models (except for OFA) scored in a similar moderate range for the SemArt dataset, although they produced lower scores than the captions generated for the COCO dataset. The average METEOR scores for the generated captions were in the low quality range suggesting the semantic similarity of the captions were not well matched.

There is a significant disconnect between what qualifies as a good general caption for someone who can use vision to engage with the image, as measured by BLEU-4 or METEOR, and the language content needed in effective accessible descriptions for BLV users. Even the most recent models cannot generate useful spatial information in the captions because the captions used in the training datasets contain little to no spatial information. If we want models to generate accessible descriptions that provide layers of the types of spatial and thematic information that our BLV participants from Study 1 found effective in helping to build mental images, then these datasets need to be developed with accessibility in mind. There will need to be additional datasets with accessible captions representing different genres of artwork on which the models can train. Previously, the problem was not having models that could generate descriptions of artwork because they were trained on natural photographs. Now, the models have advanced to perhaps support accessible description generation but model builders need to partner with assistive technology researchers and artwork curators to create the datasets with layered annotations. This should be combined with the development of better measures to evaluate the model output based on a different set of language metrics that measure the amount of spatial information in the description.

### 5.3. GPT4 Generated Descriptions

As this comparative study was already underway, several new hybrid image and LLMs were released, making the earlier captioning models appear underpowered. For example, Open AI’s GPT 4.0 [76] was released along with a browser-based demo, ChatGPT Plus, which allows users to provide an image and ask for the model to provide a caption in a particular style with a text prompt. We tested ChatGPT Plus by providing several of the same images used in Study 2 using the following prompt: *For the image above, create an appropriate description for a blind or low vision individual*. The system generated a caption for Kerry James Marshall’s painting Untitled (Painter) [57]:

*The painting features a stylized portrait of a figure with deep ebony skin. The person has prominent features, with expressive eyes and a serene facial expression. They wear large hoop earrings. Atop their head is a towering and intricate hairdo, reminiscent of an elaborately coiled bun. They’re adorned in a vibrant yellow coat with dark blue vertical stripes, paired with a striking orange, green, and white scarf draped around their neck. In their hand, they hold a paintbrush, poised above a painter’s palette filled with splotches of bright paint colors. Behind them, there’s an abstract white design resembling a paint-by-numbers portrait, with numbers labeling different sections. The background incorporates various muted colors, including dark navy and pale green, creating a contrasting backdrop to the vividly depicted central figure.*

This is a significantly longer description than the other models were able to produce and most of the description was accurate with some small errors such as the misinterpretation of the artist’s coat as a scarf. It is also important to note the lack of gender attribution to the figure. The description for our test image consisted of seven well formed sentences with rich spatial phrases (“*Atop their head…*”, “*In their hand…*”, “*poised above the painter’s palette…*”, “*Behind them*,… ”, “*The background incorporates…*”).

When ChatGPT Plus was prompted to specifically provide “*a description that focuses on spatial information for someone who cannot see Kerry James Marshall’s painting Untitled (Painter) created in 2009*”, the description produced 19 sentences and the expanded description did focus on some of the spatial information contained in the image in a more detailed language.

*“The painter is depicted in the act of creation, holding a paintbrush and palette, which adds a dynamic sense of space and action to the painting. The spatial relationship between the artist and the canvas they are working on is evident in the poised and concentrated posture, suggesting a deep connection to the act of painting. The canvas on the easel serves as a second layer of spatial interest. It portrays a scene within the scene, creating a sense of depth.”*

However, much of the remaining parts of the description were so general that it could be a part of a description for any painting.

*"The background of the painting can also be an essential aspect of its spatial composition. The choice of colors, textures, and any additional details in the background can convey a sense of depth, atmosphere, and context, enhancing the spatial experience of the artwork. Kerry James Marshall’s “Untitled (Painter)” is a masterful exploration of spatial relationships, not just in the physical layout of the composition but also in the conceptual layers that invite viewers to engage with the act of creation and the broader world of art.”*

While there is evidence that the newest versions of the GPT 4.0 powered captions can provide expanded descriptions featuring spatial language, this approach still suffers from many of the well documented issues of over generalization, inaccurate information, and a “fill in the blank” approach to generated responses. There may be ways to further refine and standardize prompts to provide more accurate and detailed spatial descriptions using a GPT4-based approach, however, this will take the collaboration of both accessibility researchers and art curatorial experts to find the right combination of AI and human in the loop cooperation to automate the process and validate/audit/monitor the results.

### 5.4. Recommendations

The findings from this set of studies provides guidance for researchers interested in improving access to artwork for BLV individuals in either an in-person museum setting or within an online collection. First, we recommend that automated accessible artwork descriptions should be structured as layered information, with a one sentence overview that provides a simple description of the content of the depicted scene along with standard information such as the artists, the title, the year and any other relevant provenance metadata. We recommend that a set of more detailed descriptions follow existing guidelines from ABS and the Coyote project with an additional level of spatial information that provides the context and the frame boundaries defined by terms such as “top/bottom”, “left/right”, “middle/center”, “corners”. The spatial description should only include information about the most salient objects in the scene, the spatial relationships between the objects, as well as other information related to distance, size, orientation, and fore/background details. Additional information about the historical, cultural, and symbolic context as well as a description of the style and form should be included separately to augment the overview and the spatial description.

Although current image captioning models (stand alone and GPT 4.0) do not meet the above accessible description recommendations, they could be refined to produce each of these types of captions. The stand alone models can help to automate the process of producing the one sentence descriptions using a model trained on artwork datasets. These types of datasets are currently limited, the annotations do not meet the above description recommendations, and creating these copyright compliant datasets will be key to improving the performance of the models. The extended captions produced by the publicly released versions of the LLMs also do not produce reliable and accurate spatial information of sufficient detail to match the quality of a human curated accessible description. The early results of this last approach appear to be promising, yet there is still much work to be done by interdisciplinary teams of computer scientists, art historians, museum curators, accessibility researchers, and BLV co-designers. Working together, these types of teams would have the knowledge and the lived experience to create image captioning models with an acceptable level of transparency, accuracy, and precision of language to be useful to museums’ accessible captioning workflows and improve access to the vast collections of artwork for the BLV community.

## 6. Limitations

As we consider the effectiveness of a model’s ability to automate the generation of accessible artwork descriptions, it is important to also consider the ethical implications of how artwork datasets are acquired to train and test an image captioning model. The dataset used in this study, SemArt, consists of older, copyright-free art paintings, which inherently carry less risk regarding copyright infringement and artist rights. It does, however, introduce known biases based on artistic depictions of race, gender, religion, and historical narratives. Incorporating other art-specific datasets into the model’s training could further enhance its performance and introduce more diversity of artistic perspectives and cultural understandings. For example, securing permission to use artwork and high-quality captions from a larger online source, such as the Getty Museum, would provide a diverse array of art from different historical periods, cultures, and artistic styles for the models to learn to generate richer descriptions. This tailored approach to training could thus lead to a more sophisticated tool for enhancing the accessibility and enjoyment of art for the BLV community.

## 7. Conclusions

This study provides evidence that several of the newest image captioning models (CLIP Prefix, BLIP, ExpansionNet, OFA) can generate short overview descriptions of artwork and have eliminated the domain gap between the training on different image types (photographs vs. artwork). For a solution that leverages the continuing advancements in LLMs, as applied to image caption generation, artwork datasets need to be developed, annotated, and released that are of sufficient size with accessible descriptions and that contain layers of both kinds of information, spatial and thematic. Collaboration between interdisciplinary teams can design accessibility supports into new models and training datasets. There is also a need to create a standard caption format designed specifically for accessible caption generation that should be based on existing research on artwork engagement.

Building on the findings of this study, an interesting avenue for future work would be to refine the training process of the most promising models. Among the assessed models, BLIP 2.0 demonstrated potential in terms of accuracy and spatial relationships in the captions. This model could be trained on more specialized datasets to enhance its performance and suitability for art description. GPT 4.0 also shows promise for generating longer, more detailed descriptions, however, the quality of the results are currently highly variable depending on the image and the quality of the prompt. Using the SemArt dataset as a starting point and supplementing spatial information within the existing captions may provide enough to train a model to produce more accessible descriptions. Training the BLIP 2.0 model on this augmented dataset could enrich its understanding of the collections’ spatial contexts, thereby potentially leading to much improved and detailed descriptions of art paintings. We also recommend the development of a different kind of caption quality metric that is designed specifically to measure the amount of spatial information in the captions, such as prepositional phrases, and language that conveys distance, size, orientation in the descriptions. This will be the direction of our future work in the automated generation of accessible descriptions.

## Figures and Tables

**Table 1:** Models by Mean Part of Speech Count per Caption.

Model	Words	Nouns	Verbs	Prepositions	Determinants
CLIP Prefix Caption	11	4	1	2	3
BLIP	10	4	1	3	3
Expansion Net	9	3	1	2	2
OFA	8	3	1	2	2

**Table 2:** Models and Generated Captions for Veronese’s “Coronation of the Virgin” (see Appendix A Figure A1).

Model	Approach	Caption
CLIP Prefix Caption	Natural Language supervision contrastive learning	A painting of three people sitting on a couch.
BLIP	Vision-Language Pre-training (VLP)	A painting of a woman being comforted by two men.
Expansion Net	Block Static Expansion	A painting of a group of people standing in a room.
OFA	CNN and Transformer	A painting of a group of people.

**Table 3:** Generated Captions Metrics (Models by Datasets).

	COCO		SemArt	
Model	BLEU-4	METEOR	BLEU-4	METEOR
CLIP Prefix	33.5	27.5	39.2	8.6
BLIP	40.4	31.2	33.6	8.7
Expansion Net	42.7	30.6	40.2	7.5
OFA	44.9	32.5	20.4	8.2

**Figure A1:**
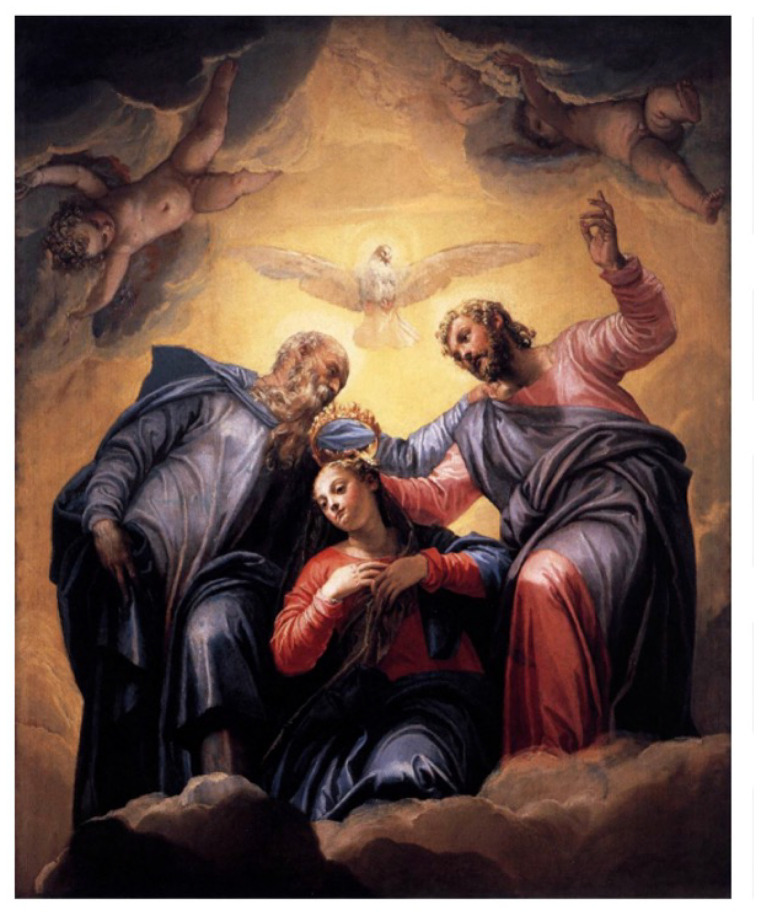
Paolo Veronese “Coronation of the Virgin”. 1555 Oil on canvas, 200 × 170 cm San Sebastiano, Venice [77].

## Data Availability

The data presented in this study are available on request from the corresponding author. The data are not publicly available due to restrictions based on the institutional review board.

## Abbreviations

The following abbreviations are used in this manuscript:

BLV	Blindness and Low Vision
LLM	Large Language Model
ABS	Art Beyond Sight
MCA	Chicago Museum of Contemporary Art
HRNN	Hierarchical Recurrent Neural Networks
CNN	Convolutional Neural Netowrks
GPT	Generative Pre-trained Transformer
VLP	Vision-Language-Pre-training
VQA	Vision-Question-Answering
OFA	One For All
NLP	Natural Language Processing
POS	Parts of Speech

## Appendix A

**Table A1:** Participant Demographics, Etiology, and Residual Vision.

PID	Gender	Vision Loss Cause	Residual Vision	Onset
P1	Female	Leber’s Congenital Amaurosis	Light/Dark Perception	present at birth
P2	Male	Retinopathy of Prematurity	Light/Dark Perception	30 years
P3	Female	Bilateral Retna Blastoma	None	3.5 years
P4	Male	Retinopathy of Prematurity	None	present at birth
P5	Female	Marfan’s syndrome/retinal detachment	None	4 years
P6	Female	Retinopathy of prematurity	None	present at birth
P7	Male	Glaucoma	Light/Dark Perception	present at birth
P8	Female	Unknown	Light/Dark Perception	38 years
P9	Male	Retinal Detachment	None	31 years
P10	Male	Glaucoma	Light/Dark Perception	30 years
P11	Female	Retinopathy of Prematurity	Light/Dark Perception	present at birth
